# Baseline imbalances in a real-world study of TKI plus radiotherapy for HCC with PVTT: Implications for interpreting survival outcomes

**DOI:** 10.1016/j.bj.2026.101022

**Published:** 2026-08-06

**Authors:** Siyuan Zhu, Peiru Zhang

**Affiliations:** The Second Affiliated Hospital of Fujian Medical University, Quanzhou, Fujian, China

To the Editor,

We read with great interest the article by Huang and colleagues on TKI plus radiotherapy for HCC with PVTT^1^. The 15.0-month median survival in the combination group is encouraging, and we commend the authors for their transparency about study limitations including lack of randomization.

However, given the retrospective and non-randomized nature of this study, we wish to respectfully highlight the substantial baseline imbalances between treatment groups that are evident in Table 1, as these differences may influence the interpretation of the comparative survival outcomes.

Although multivariable adjustment was applied, residual confounding from severe baseline disparities may contribute to the reported survival benefit. This study supports prospective randomized trials, and we suggest future trials stratify by ALBI grade at randomization. The promising results warrant cautious interpretation, as part of the benefit may stem from favorable baseline characteristics.

## Declaration of competing interest

None declared.
